# Clinical characteristics of airway impairment assessed by impulse oscillometry in patients with chronic obstructive pulmonary disease: findings from the ECOPD study in China

**DOI:** 10.1186/s12890-023-02311-z

**Published:** 2023-02-03

**Authors:** Lifei Lu, Jieqi Peng, Fan Wu, Huajing Yang, Youlan Zheng, Zhishan Deng, Ningning Zhao, Cuiqiong Dai, Shan Xiao, Xiang Wen, Jianwu Xu, Xiaohui Wu, Kunning Zhou, Pixin Ran, Yumin Zhou

**Affiliations:** 1 State Key Laboratory of Respiratory Disease, National Center for Respiratory Medicine, National Clinical Research Center for Respiratory Disease, Guangzhou Institute of Respiratory Health, The First Affiliated Hospital of Guangzhou Medical University, 151 Yanjiang Road, Guangzhou, China; 2 Guangzhou Laboratory, Guangzhou, China

**Keywords:** Impulse oscillometry, Airway impairment, Chronic obstructive pulmonary disease, Computed tomography, Spirometry

## Abstract

**Background:**

The role of airway impairment assessed by impulse oscillometry (IOS) in patients with chronic obstructive pulmonary disease (COPD) remains unclear. Therefore, this study aimed to analyze the proportion and clinical characteristics of airway impairment assessed by IOS across COPD severities, and explore whether airway impairment is a subtype of COPD.

**Methods:**

This study was based on cross-sectional data from the ECOPD cohort in Guangdong, China. Subjects were consecutively recruited from July 2019 to August 2021. They filled out questionnaires and underwent lung function tests, IOS and computed tomography (CT). COPD was defined as post-bronchodilator forced expiratory volume in one second/forced vital capacity < lower limit of normal (LLN). Meanwhile, airway impairment was defined as IOS parameters > upper limit of normal or < LLN. On the one hand, Poisson regression was employed to analyze the associations between acute exacerbations of COPD (AECOPD) in the previous year and airway impairment. On the other hand, logistic regression was used to assess differences in CT imaging between patients with IOS parameters’ abnormalities and patients with normal IOS parameters.

**Results:**

768 COPD subjects were finally enrolled in the study. The proportion of airway impairment assessed by R_5_, R_20_, R_5_–R_20_, X_5_, AX, and F_res_ was 59.8%, 29.7%, 62.5%, 52.9%, 60.9% and 67.3%, respectively. Airway impairment assessed by IOS parameters (R_5_, R_5_–R_20_, X_5_, AX, and F_res_) in patients with COPD was present across all severities of COPD, particularly in GOLD 3–4 patients. Compared with patients with normal IOS parameters, patients with IOS parameters’ abnormalities had more respiratory symptoms, more severe airway obstruction and imaging structural abnormalities. Patients with IOS parameters’ abnormalities assessed by R_5_ [risk ratio (RR): 1.58, 95% confidential interval (CI): 1.13–2.19, P = 0.007], R_5_–R_20_ [RR: 1.73, 95%CI: 1.22–2.45, P = 0.002], X_5_ [RR: 2.11, 95%CI: 1.51–2.95, P < 0.001], AX [RR: 2.20, 95%CI: 1.53–3.16, P < 0.001], and F_res_ [RR: 2.13, 95%CI: 1.44–3.15, P < 0.001] had a higher risk of AECOPD in the previous year than patients with normal IOS parameters.

**Conclusions:**

Airway impairment assessed by IOS may be a subtype of COPD. Future studies are warranted to identify the underlying mechanisms and longitudinal progression of airway impairment.

**Supplementary Information:**

The online version contains supplementary material available at 10.1186/s12890-023-02311-z.

## Introduction

Chronic obstructive pulmonary disease (COPD) is a common, preventable and treatable disease characterized by irreversible airflow obstruction. It has become a worldwide public health challenge owing to its high prevalence, disability, and mortality [1]. In 2019, the global prevalence of COPD in individuals aged 30–79 years, as defined by the Global Initiative for Chronic Obstructive Lung Disease (GOLD) standard, was 10.3% [95% confidential interval (CI) 8.2–12.8] or 391.9 million (95% CI 312.6–487.9) [2]. As is well documented, small airways are the primary sites of airflow obstruction in COPD [3]. Histological data suggested that small airway inflammatory lesions may lead to a decrease in airway diameter, increased airway impedance, and eventually progress to airway impairment [4].

Impulse oscillometry (IOS) is gaining prominence worldwide and plays a crucial role in patients with asthma as it captures its variability [5]. In COPD patients, lung function is typically used to detect airway obstruction in clinical practice. However, compared with lung function, IOS only requires patients to breathe calmly to measure airway impedance and more accurately detect large and small airway functions [6]. Previous study has identified the number of small airway dysfunction (SAD) diagnosed by only IOS but lung function not was nearly twice than that by only lung function but IOS not in normal subjects [7], indicating that IOS could supplement information that lung function cannot obtain. Other studies have determined that airway impairment detected via IOS were associated with impaired lung function and airway lesions [8–10]. Indeed, IOS parameters increase with the severities of COPD [8, 11, 12]. Nevertheless, the proportion of airway impairment assessed by IOS and the clinical characteristics of airway impairment in patients with COPD remain unknown.

There were two primary objectives in this study. Firstly, we aimed to explore the proportion of airway impairment assessed by IOS based on Chinese IOS predictive values [> upper limit of normal (ULN)/ < lower limit of normal (LLN)] across COPD severities. Secondly, clinical characteristics of airway impairment in patients with COPD were assessed to analyze whether airway impairment was a COPD phenotype.

## Methods

### Study design and participants

The ECOPD cohort was a prospective, observational, population-based cohort study in China. The baseline, cross-sectional data of COPD patients were collected from the ECOPD study. The ECOPD cohort research design has already been published [13]. In short, subjects were continuously recruited between July 2019 and August 2021 in Guangzhou, Shaoguan, and Heyuan, Guangdong province, China.

Subjects in this study were 40–80 years old, filled out questionnaires, and underwent pre-bronchodilator IOS, pre-bronchodilator lung function tests, post-bronchodilator lung function tests and computed tomography (CT). COPD was defined as a post-bronchodilator forced expiratory volume in one second (FEV_1_)/forced vital capacity (FVC) < LLN, and was based on a multicenter Chinese population spirometry predicted values study [14]. Sensitivity analysis adopted the criterion of post-bronchodilator FEV_1_/FVC < 0.70 for the diagnosis of COPD. The exclusion criteria were as follows: (I) aged < 40 years or > 80 years; (II) respiratory infection or exacerbation in the 4 weeks prior to screening; (III) heart attack (myocardial infarction, malignant arrhythmia) in the past 3 months; (IV) hospitalized for heart disease within the past 1 month; (V) chest, abdomen, or eye surgery in the past 3 months; (VI) previous lobectomy; (VII) malignant tumors newly discovered and being treated; (VIII) receiving anti-tuberculosis drug treatment or active pulmonary tuberculosis; (IX) history of mental disorders, auditory hallucinations, visual hallucinations, or taking antipsychotic drugs; (X) history of cognitive disorders, including dementia or cognitive disorders; (XI) history of high paraplegia; and (XII) pregnant or lactating women.

This study was conducted in accordance with the Declaration of Helsinki and approved by The Ethics Committee of the First Affiliated Hospital of Guangzhou Medical University (Approval Number 2018–53). All participants signed the appropriate informed consent prior to their inclusion.

### Questionnaires

The questionnaire in this study was revised in accordance with the Chinese COPD epidemiology study, including smoking status, smoking index, history of occupational exposure and family history of respiratory diseases [15, 16]. History of occupational exposure was defined as occupational exposure for more than 1 year in a participant's lifetime. Biomass exposure was defined as cooking or heating using biomass (mainly wood, crop residues, charcoal, grass, and dung) for more than 1 year. Family history of respiratory diseases was defined as having parents, siblings, and children with respiratory diseases (chronic bronchitis, emphysema, asthma, COPD, cor pulmonale, bronchiectasis, lung cancer, interstitial lung disease, or obstructive sleep apnea–hypopnea syndrome). Chronic respiratory symptoms included cough, phlegm, wheeze, and dyspnea. Symptom severities was assessed by the modified Medical Research Council Dyspnea Scale (mMRC) score and COPD Assessment Test (CAT) [17]. Acute exacerbations of COPD (AECOPD) were defined as the presence or exacerbation of at least two of the following symptoms: cough, sputum production, purulent sputum, wheezing, and dyspnea lasting at least 48 h, after excluding left–right cardiac dysfunction, pulmonary embolism, pneumothorax, pleural effusion, arrhythmia, and other diseases [18]. Chronic bronchitis was defined as chronic cough and expectoration for at least 3 months a year for two consecutive years [19].

### Lung function

According to the lung function criteria recommended by the American Thoracic Society/European Respiratory Society (ERS) [20], trained physicians carried out daily calibration verification of lung function (CareFusion, Yorba Linda, CA, USA) at low, medium, and high flow. The acceptable reproducibility criterion was a single test consisting of no hesitation at the onset of expiration and an extrapolated volume of < 5% FVC or 150 ml. The largest and second-largest values for FEV_1_ and FVC had to be within 150 ml, and the experiments were at least performed in triplicates. Subjects performed a post-bronchodilator lung function test after inhaling 400 µg of salbutamol (Ventolin, Glaxo-SmithKline) via a 500-ml spacer for 20 min. The criteria for the severity of COPD were as follows: GOLD 1, 80% ≤ FEV_1_% predicted; GOLD 2, 50% ≤ FEV_1_% predicted < 80%; GOLD 3, 30% ≤ FEV_1_% predicted < 50%; GOLD 4, FEV_1_% predicted < 30% [1].

### Impulse oscillometry (IOS)

Respiratory resistance and reactance were measured using IOS (Master Screen IOS, Hochberg Germany). Subjects did not receive bronchodilators for 72 h before the IOS test. According to the ERS recommendation [21], the operator gently pressed the subject's cheeks with both hands to avoid cheek vibration affecting the accuracy of measurement. The subject sat upright, clipped into the nose clip and carried out tidal breath for at least 30 s. Cough, swallowing, and air leakage were avoided during this period. The quality control was the within-session coefficient of variation of resistance at 5 Hz (R_5_) less than 10% [22]. R_5_ indicated total respiratory resistance; resistance at 20 Hz (R_20_) represented central airway resistance; the difference between R_5_ and R_20_ (R_5_–R_20_), reflected peripheral airway resistance; reactance at 5 Hz (X_5_) was a measure of the stiffness of the entire system; resonance frequency (F_res_), where E_rs_ and I_rs_ made equal and opposite contributions to impedance and reactance, was a sensitive index reflecting increase resistance; AX, the area under X_5_ and F_res_, reflected the comprehensive index of reactance [23]. The criteria for airway impairment were based on a multicenter Chinese population IOS predictive value [24]. Patients with COPD were divided into six IOS parameters’ abnormalities groups according to the following criteria: R_5_ > ULN, R_20_ > ULN, R_5_-R_20_ > ULN, X_5_ < LLN, AX > ULN, F_res_ > ULN.

### Computed tomography

Two multidetector-row CT scanner (Siemens Definition AS Plus 128-slicers and United-imaging uCT 760 128-slicers) was utilized for high-resolution CT. Percent emphysema was defined as the total percentage of both lungs with attenuation values less than -950 Hounsfield units on inspiratory images (inspiratory LAA_-950_). Meanwhile, percent gas trapping was defined as the total percentage of both lungs with attenuation values less than -856 Hounsfield units on expiratory images (expiratory LAA_-856_). Lastly, emphysema > 5% and air trapping > 20% were defined as abnormal lesions [25].

### Statistical analysis

Statistical analyses were performed using SPSS statistics version 26.0 (IBM Corp. Armonk, NY, USA). Continuous variables following a normal distribution were expressed as mean (standard deviation), and continuous variables not following a normal distribution were expressed as median [interquartile range (IQR)]. The Student’s t-test was used to compare differences between the IOS parameters’ abnormalities group with the group with the normal IOS parameters for normal continuous variables, while the Wilcoxon rank-sum test was utilized for non-normal continuous variables, and Fisher’s exact or Chi-squared test for categorical variables. After adjusting for age, sex, body mass index (BMI), smoking index, smoking status, family history of respiratory diseases, occupational exposures, biomass exposure, and history of asthma, logistic regression was used to assess differences in CT imaging between the group with IOS parameters’ abnormalities and group with normal IOS parameters. The associations between AECOPD in the previous year and airway impairment were assessed by Poisson regression. Sensitivity analysis used post-bronchodilator FEV_1_/FVC < 0.70 to define COPD. P < 0.05 was considered statistically significant.

## Results

### Patient characteristics

**Fig. 1:**
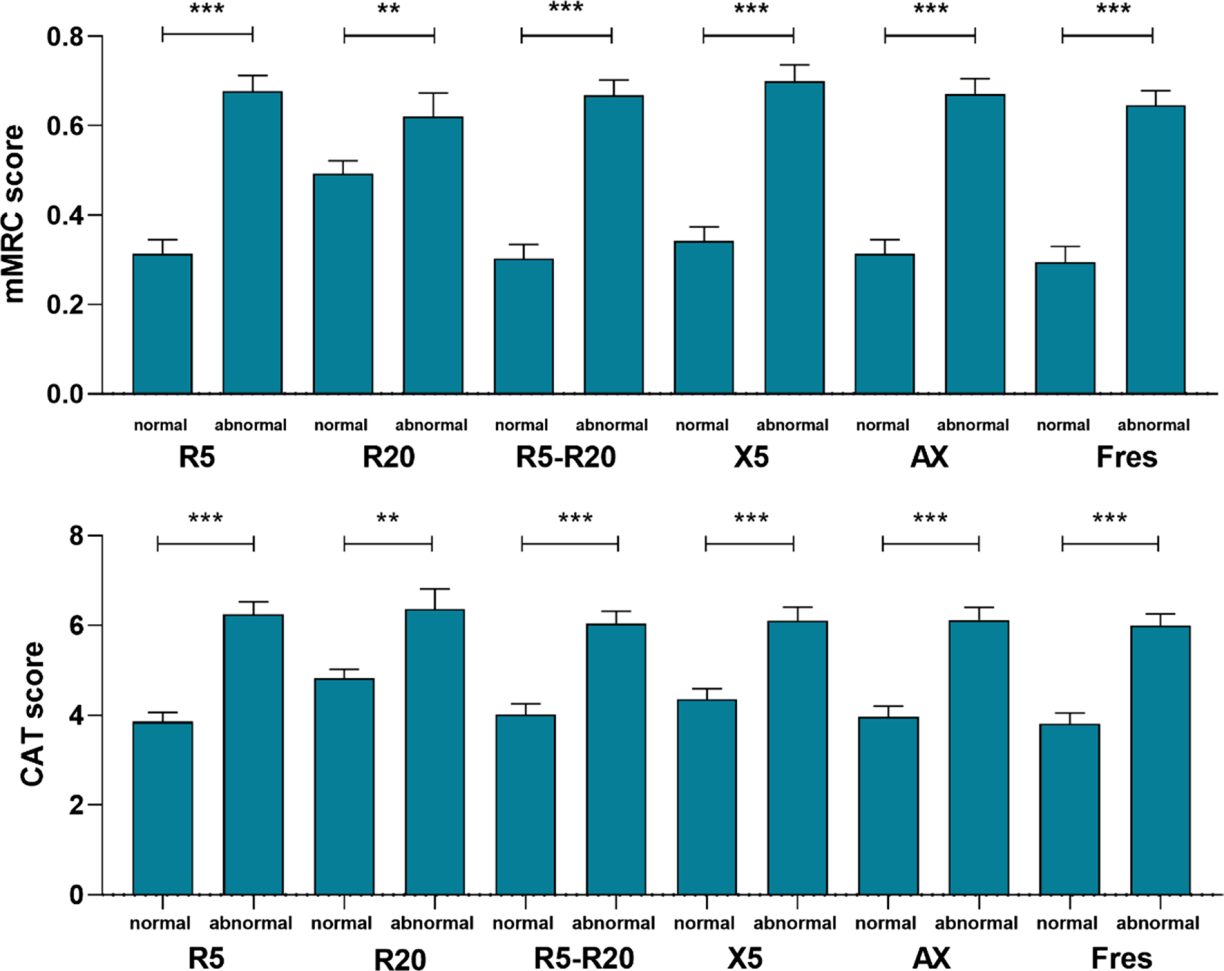
mMRC and CAT scores between airway impairment group and non-airway impairment group in patients with chronic obstruction pulmonary disease, datas were shown as mean (SE) ** means < 0.01, *** means < 0.001

The proportion of airway impairment assessed by R_5_, R_20_, R_5_-R_20_, X_5_, AX, and F_res_ in COPD patients was 59.8%, 29.7%, 62.5%, 52.9%, 60.9% and 67.3%, respectively. Compared with patients with normal IOS parameters, patients with IOS parameters’ abnormalities groups (R_5_, R_5_-R_20_, X_5_, AX, and F_res_) had more respiratory symptoms (cough, phlegm, wheeze, and dyspnea), asthma, more emphysema and air trapping. However, there was no significant difference in respiratory symptoms and emphysema between patients with normal IOS parameters and patients with IOS parameters’ abnormalities assessed by R_20_. Compared with patients with normal IOS parameters, patients with IOS parameters’ abnormalities (R_5_–R_20_, X_5_, AX, and F_res_) were older and had a higher proportion of ever-smokers, whereas patients with IOS parameters’ abnormalities (R_5_–R_20_ and F_res_) had lower BMI and higher smoking index. Patients with IOS parameters’ abnormalities assessed by R_20_ had more occupational exposures and F_res_ had a higher proportion of chronic bronchitis. Besides, there was no statistical difference in gender and family history of respiratory diseases between patients with normal IOS parameters and patients with IOS parameters’ abnormalities (Table 1). Contrastingly, patients with IOS parameters’ abnormalities had more impaired lung function and severe airway obstruction (Table 2) and higher mMRC and CAT scores (Fig. 1) than patients with normal IOS parameters.

**Table 1:** Baseline characteristics of airway impairment assessed by IOS in patients with chronic obstruction pulmonary disease

	R_5_		R_20_		R_5_-R_20_		X_5_		AX		F_res_	
Value	≤ ULN	> ULN	≤ ULN	> ULN	≤ ULN	> ULN	≥ LLN	< LLN	≤ ULN	> ULN	≤ ULN	> ULN
Number, n (%)	309 (40.2)	459 (59.8)	540 (70.3)	228 (29.7)	288 (37.5)	480 (62.5)	362 (47.1)	406 (52.9)	300 (39.1)	468 (60.9)	251 (32.7)	517 (67.3)
Age	63.71 (7.26)	64.56 (7.33)	64.30 (7.07)	64.02 (7.85)	62.74 (7.15)	65.10 (7.26)*	63.03 (7.18)	65.28 (7.27)*	62.91 (7.18)	65.05 (7.27)*	62.17 (7.19)	65.21 (7.16)*
Male, n (%)	283 (91.6)	414 (90.2)	491 (90.9)	206 (90.4)	254 (88.2)	443 (92.3)	335 (92.5)	362 (89.2)	277 (92.3)	420 (89.7)	225 (89.6)	472 (91.3)
BMI	22.15 (2.93)	21.87 (3.38)	21.84 (3.12)	22.32 (3.39)	22.35 (3.09)	21.76 (3.26) ^†^	22.13 (2.92)	21.86 (3.45)	22.10 (3.02)	21.91 (3.32)	22.44 (3.16)	21.76 (3.21) ^†^
Smoking statue, n (%)												
Never	41 (13.3)	60 (13.1)	68 (12.6)	33 (14.5)	46 (16.0)	55 (11.5)	45 (12.4)	56 (13.8)	39 (13.0)	62 (13.2)	40 (15.9)	61 (11.8)
Ever	72 (23.3)	154 (33.6) ^†^	150 (27.8)	76 (33.3)	63 (21.9)	163 (34.0) ^†^	86 (23.8)	140 (34.5) ^†^	69 (23.0)	157 (33.5) ^†^	57 (22.7)	169 (32.7) ^†^
Current	196 (63.4)	245 (53.4) ^†^	322 (59.6)	119 (52.2)	179 (62.2)	262 (54.6) ^†^	231 (63.8)	210 (51.7) ^†^	192 (64.0)	249 (53.2) ^†^	154 (61.4)	287 (55.5)
Pack-years	34.14 (29.26)	38.38 (33.18)	36.63 (30.92)	36.80 (33.59)	33.68 (30.03)	38.48 (32.58) ^†^	34.87 (29.81)	38.28 (33.27)	34.35 (29.47)	38.17 (33.01)	32.95 (29.08)	38.49 (32.79) ^†^
Family history of respiratory diseases, n (%)	54 (17.5)	92 (20.0)	99 (18.3)	47 (20.6)	54 (18.8)	92 (19.2)	67 (18.5)	79 (19.5)	54 (18.0)	92 (19.7)	42 (16.7)	104 (20.1)
Occupational exposures, n (%)	84 (27.2)	124 (27.0)	132 (24.4)	76 (33.3) ^†^	77 (26.7)	131 (27.3)	112 (30.9)	96 (23.6) ^†^	82 (27.3)	126 (26.9)	66 (26.3)	142 (27.5)
Biomass exposure, n (%)	110 (35.6)	178 (38.8)	204 (37.8)	84 (36.8)	102 (35.4)	186 (38.8)	132 (36.5)	156 (38.4)	107 (35.7)	181 (38.7)	87 (34.7)	201 (38.9)
History of CB, n (%)	18 (5.8)	41 (8.9)	42 (7.8)	17 (7.5)	19 (6.6)	40 (8.3)	25 (6.9)	34 (8.4)	19 (6.3)	40 (8.5)	12 (4.8)	47 (9.1) ^†^
History of asthma, n (%)	2 (0.6)	20 (4.4) ^†^	11 (2.0)	11 (4.8) ^†^	2 (0.7)	20 (4.2) ^†^	1 (0.3)	21 (5.2)*	1 (0.3)	21 (4.5) ^†^	1 (0.4)	21 (4.1) ^†^
Drug treatment, n (%)	126 (40.8)	249 (54.2)*	255 (47.2)	120 (52.6)	114 (39.6)	261 (54.4)*	151 (41.7)	224 (55.2)*	123 (41.0)	252 (53.8) ^†^	98 (39.0)	277 (53.6)*
*Clinical symptoms, (n%)*												
Cough	109 (35.3)	218 (47.5) ^†^	228 (42.2)	99 (43.4)	99 (34.4)	228 (47.5)*	133 (36.7)	194 (47.8) ^†^	106 (35.3)	221 (47.2) ^†^	82 (32.7)	245 (47.4)*
Phlegm	127 (41.1)	268 (58.4)*	272 (50.4)	123 (53.9)	119 (41.3)	276 (57.5)*	157 (43.4)	238 (58.6)*	122 (40.7)	273 (58.3)*	97 (38.6)	298 (57.6)*
Wheeze	32 (10.4)	111 (24.2)*	91 (16.9)	52 (22.8)	32 (11.1)	111 (23.1)*	40 (11.0)	103 (25.4)*	31 (10.3)	112 (23.9)*	26 (10.4)	117 (22.6)*
Dyspnea	87 (28.2)	237 (51.7)*	218 (40.4)	106 (46.7)	77 (26.7)	247 (51.6)*	104 (28.7)	220 (54.3)*	84 (28.0)	240 (51.4)*	64 (25.5)	260 (50.4)*
GOLD stage, n (%)												
GOLD 1	195 (63.1)	82 (17.9)*	225 (41.7)	52 (22.8)*	185 (64.2)	92 (19.2)*	207 (57.2)	70 (17.2)*	193 (64.3)	84 (17.9)*	173 (68.9)	104 (20.1)*
GOLD 2	107 (34.6)	274 (59.7)*	242 (44.8)	139 (61.0)*	100 (34.7)	281 (58.5)*	144 (39.8)	237 (58.4)*	102 (34.0)	279 (59.6)*	76 (30.3)	305 (59.0)*
GOLD 3	7 (2.3)	86 (18.7)*	62 (11.5)	31 (13.6)*	3 (1.0)	90 (18.8)*	11 (3.0)	82 (20.2)*	5 (1.7)	88 (18.8)*	2 (0.8)	91 (17.6)*
GOLD 4	0 (0)	17 (3.7)*	11 (2.0)	6 (2.6)*	0 (0)	17 (3.5)*	0 (0)	17 (4.2)*	0 (0)	17 (3.6)*	0 (0)	17 (3.3)*
Emphysema on CT, (%)	59 (19.1)	170 (37.0)*	163 (30.2)	66 (28.9)	44 (15.3)	185 (38.5)*	77 (21.3)	152 (37.4)*	53 (17.7)	176 (37.6)*	38 (15.1)	191 (36.9)*
Air trapping on CT, (%)	139 (45.0)	310 (67.5)*	305 (56.5)	144 (63.2)	118 (41.0)	331 (69.0)*	166 (45.9)	283 (69.7)*	129 (43.0)	320 (68.4)*	98 (39.0)	351 (67.9)*

Datas are presented as the mean (standard deviation) or median (interquartile range) and were analyzed by Student’s t-test or Wilcoxon’s rank-sum test.; BMI, body mass index; MMEF, maximum mid expiratory flow, MMEF; FEV_1_, forced expiratory volume in one second; CT, computed tomography. CB, chronic bronchitis

^†^P < 0.05; *P < 0.001

**Table 2:** Post-bronchodilator lung function and pre-bronchodilator IOS parameters of airway impairment in patients with chronic obstruction pulmonary disease

	R_5_		R_20_		R_5_-R_20_		X_5_		AX		F_res_	
Value	≤ ULN	> ULN	≤ ULN	> ULN	≤ ULN	> ULN	≤ ULN	> ULN	≤ ULN	> ULN	≤ ULN	> ULN
Spirometry												
FEV_1_, L	2.30 (0.51)	1.72 (0.56)*	2.01 (0.62)	1.82 (0.56)*	2.31 (0.51)	1.74 (0.56)*	2.28 (0.54)	1.66 (0.52)*	2.34 (0.49)	1.70 (0.55)*	2.38 (0.49)	1.74 (0.55)*
FEV_1_, %pred	83.20 (14.33)	63.72 (17.47)*	73.30 (19.34)	67.43 (17.04)*	84.21(13.36)	63.96 (17.58)*	81.60 (15.12)	62.61 (17.33)*	84.23 (13.52)	63.43 (17.28)*	85.63(13.19)	64.73 (17.38)*
FVC, L	3.69 (0.73)	3.14 (0.73)*	3.43 (0.78)	3.21 (0.73)*	3.69 (0.74)	3.17 (0.73)*	3.71 (0.73)	3.06 (0.67)*	3.76 (0.70)	3.11 (0.71)*	3.77 (0.71)	3.17 (0.72)*
FVC, %pred	105.11(15.81)	91.66 (16.40)*	98.40 (17.77)	93.94 (16.27) ^†^	106.15 (15.12)	91.63 (16.47)*	104.80 (15.43)	90.19 (16.23)*	106.57(15.14)	90.99 (16.07)*	107.25 (14.97)	92.14 (16.41)*
FEV_1_/FVC, L	62.24 (6.10)	54.19 (10.42)*	57.92 (9.64)	56.26 (10.00) ^†^	62.72 (5.90)	54.25 (10.25)*	61.27 (6.84)	54.00 (10.67)*	62.38 (5.93)	54.25 (10.41)*	63.18 (5.42)	54.64 (10.18)*
FEF_50_, %pred	44.26 (14.45)	28.69 (13.93)*	36.10 (16.56)	32.20 (14.47) ^†^	45.25 (14.30)	28.78 (13.73)*	42.67 (15.20)	28.04 (13.46)*	45.08 (14.17)	28.47 (13.68)*	46.81 (13.91)	29.21 (13.72)*
FEF_75_, %pred	37.09 (14.27)	28.89 (12.28)*	32.47 (13.95)	31.49 (13.15)	37.61 (14.43)	28.94 (12.18)*	36.18 (14.39)	28.60 (12.02)*	37.65 (14.65)	28.69 (11.84)*	37.95 (14.09)	29.39 (12.63)*
MMEF, %pred	41.61 (12.79)	29.04 (12.63)*	34.85 (14.34)	32.28 (13.39) ^†^	42.63 (12.99)	28.98 (12.15)*	40.35 (13.41)	28.49 (12.26)*	42.46 (12.95)	28.74 (12.08)*	43.70 (12.58)	29.44 (12.36)*
IOS												
R_5_, kPa/L/s	0.26 (0.23–0.29)	0.42 (0.37–0.51)*	0.31 (0.26–0.40)	0.45 (0.38–0.54)*	0.27 (0.24–0.32)	0.41 (0.34–0.50)*	0.28 (0.24–0.34)	0.42 (0.36–0.52)*	0.27 (0.23–0.32)	0.42 (0.35–0.51)*	0.27 (0.23–0.32)	0.40 (0.33–0.49)*
R_20_, kPa/L/s	0.23 (0.20–0.25)	0.30 (0.26–0.34)*	0.25 (0.22–0.27)	0.33 (0.31–0.38)	0.25 (0.22–0.30)	0.27 (0.24–0.32)*	0.25 (0.22–0.30)	0.28 (0.25–0.33)*	0.24 (0.21–0.29)	0.28 (0.25–0.32)*	0.25 (0.22–0.30)	0.27 (0.24–0.32)*
R_5_–R_20_, kPa/L/s	0.03 (0.01–0.05)	0.13 (0.08–0.19)*	0.06 (0.03–0.13)	0.10 (0.05–0.18)*	0.02 (0.01–0.04)	0.12 (0.08–0.19)*	0.04 (0.02–0.06)	0.14 (0.09–0.20)*	0.03 (0.01–0.04)	0.13 (0.08–0.19)*	0.02 (0.01–0.04)	0.11 (0.07–0.18)*
X_5_, kPa/L/s	− 0.09 ( − 0.11–0.07)	− 0.17 ( − 0.24–0.12)*	− 0.11 ( − 0.17–0.08)	− 0.16 ( − 0.24–0.10)*	− 0.09 ( − 0.11–0.07)	− 0.16 ( − 0.24–0.11)*	− 0.09 ( − 0.10–0.07)	− 0.18 ( − 0.25–0.14)*	− 0.08( − 0.10–0.07)	− 0.17 ( − 0.24–0.12)*	− 0.08 ( − 0.10–0.07)	− 0.15 ( − 0.23–0.11)*
AX, kPa/L	0.24 (0.15–0.40)	1.27 (0.65–2.27)*	0.49 (0.22–1.21)	1.09 (0.42–2.35)*	0.22 (0.13–0.33)	1.21 (0.64–2.21)*	0.25 (0.15–0.41)	1.48 (0.81–2.37)*	0.22 (0.14–0.30)	1.23 (0.70–2.24)*	0.19 (0.12–0.27)	1.09 (0.58–2.07)*
F_res_, HZ	12.33 (9.63–15.24)	21.53 (17.62–25.27)*	16.00 (11.64–21.40)	20.47 (14.96–26.44)*	11.39 (9.32–13.74)	21.43 (17.50–25.03)*	12.86 (9.80–15.90)	21.75 (17.96–25.65)*	11.44 (9.37–13.47)	21.60 (18.05–25.24)*	10.66 (9.17–12.81)	20.91 (16.85–24.75)*

Datas are presented as the mean (standard deviation) or median (interquartile range) and were analyzed by Student’s t-test or Wilcoxon’s rank-sum test; IOS, impulse oscillometry; R_5_, resistance at 5 Hz; R_20_, resistance at 20 Hz; R_5_–R_20_, difference from R5 to R20; X_5_, reactance at 5 Hz; F_res_, resonant frequency. FEV_1_, forced expiratory volume in one second; FVC, forced vital capacity; MMEF, maximal mid-expiratory flow; FEF_50_, forced expiratory flow 50%; FEF_75_, forced expiratory flow 75%

^†^P < 0.05; *P < 0.001

### The proportion of airway impairment in different GOLD grades

**Fig. 2:**
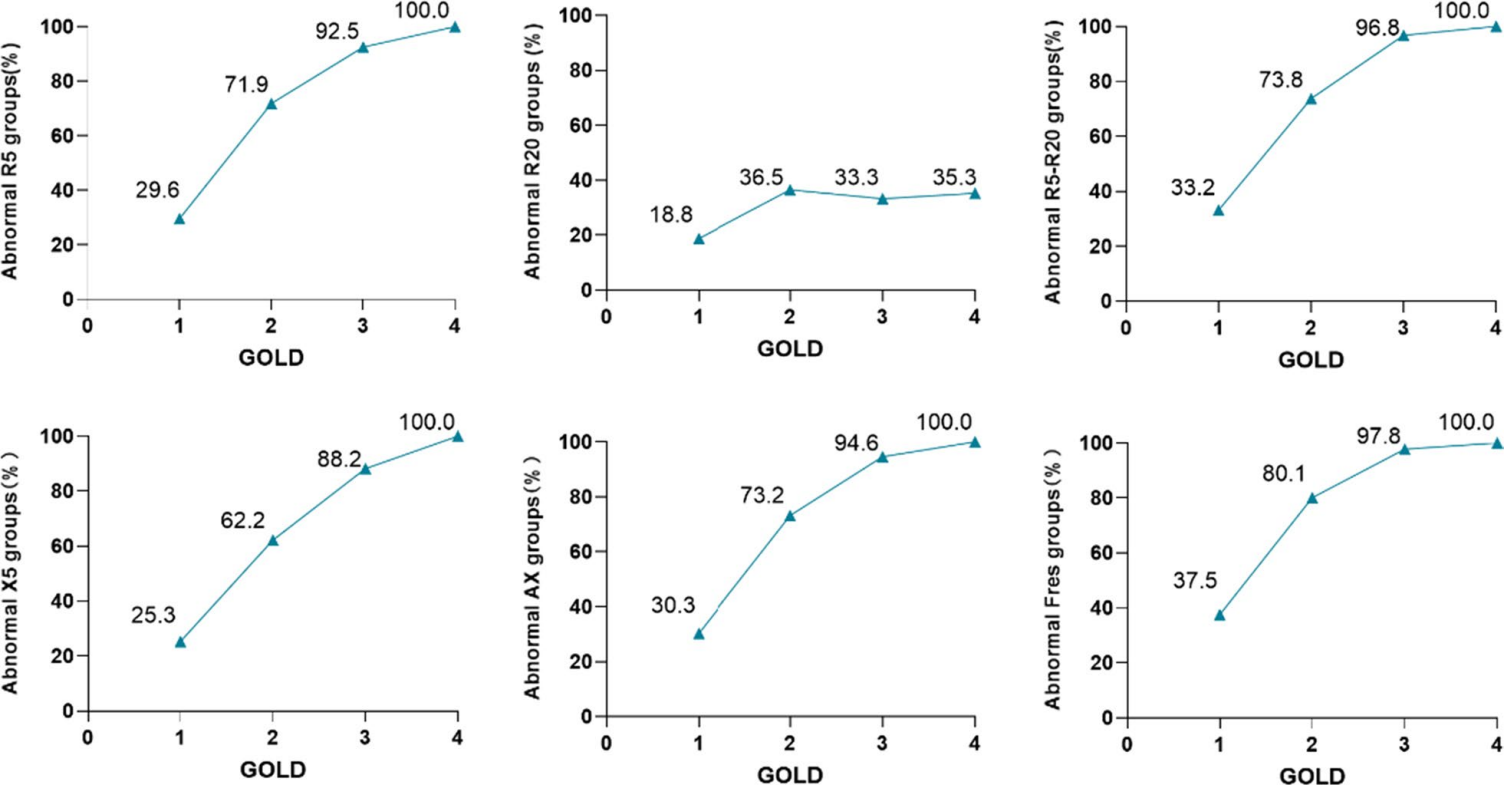
Proportion of airway impairment in COPD patients with different GOLD grades

Airway impairment was present across patients with all severities of COPD, particularly in COPD patients with GOLD 3–4. The proportion of airway impairment (R_5_, R_5_-R_20_, X_5_, AX, and F_res_) progressively increased with an increase in the severities of COPD. For instance, the proportion of the airway impairment was the lowest in patients with GOLD 1 and higher in GOLD 2–3, and airway impairment was all identified in COPD patients with GOLD 4. There was a significant difference in airway impairment among COPD patients with GOLD 1–3 but no difference in COPD patients with GOLD 3–4. However, the proportion of airway impairment assessed by R_20_ did not increase significantly with the severities of COPD (GOLD 1, 18.8%; GOLD 2, 36.5%; GOLD 3, 33.3%; GOLD 4, 35.3%) (Fig. 2).

### Risk of acute exacerbations of COPD in the previous year and airway impairment

Compared with patients with normal IOS parameters, patients with IOS parameters’ abnormalities assessed by R_5_ [risk ratio (RR): 1.58, 95% CI: 1.13–2.19, P = 0.007], R_5_–R_20_ [RR: 1.73, 95%CI: 1.22–2.45, P = 0.002], X_5_ [RR: 2.11, 95%CI: 1.51–2.95, P < 0.001], AX [RR: 2.20, 95%CI: 1.53–3.16, P < 0.001], and F_res_ [RR: 2.13, 95%CI: 1.44–3.15, P < 0.001] were at a significantly higher risk of AECOPD in the previous year (Table 3).

**Table 3:** Associations between acute exacerbations of COPD in the previous year and airway impairment in patients with chronic obstruction pulmonary disease

AECOPD in the previous year	With airway impairment	Without airway impairment	Risk ratio (95%CI) ^#^	P value
Total—per patient-year	R_5_ > ULN (n = 459)	R_5_ ≤ ULN (n = 308)		
	0.18 (0.07)	0.11 (0.05) ^†^	1.58 (1.13–2.19)	**0.007**
Total—per patient-year	R_20_ > ULN (n = 228)	R_20_ ≤ ULN (n = 539)		
	0.16 (0.06)	0.17 (0.07)	0.93 (0.67—1.29)	0.661
Total—per patient-year	R_5_-R_20_ > ULN (n = 480)	R_5_-R_20_ ≤ ULN (n = 287)		
	0.19 (0.07)	0.11 (0.04) ^†^	1.73 (1.22—2.45)	**0.002**
Total—per patient-year	X_5_ < LLN (n = 406)	X_5_ ≥ LLN (n = 361)		
	0.19 (0.07)	0.09 (0.04) ^†^	2.11 (1.51–2.95)	** < 0.001**
Total—per patient-year	AX > ULN (n = 468)	AX ≤ ULN (n = 299)		
	0.18 (0.07)	0.08 (0.03) ^†^	2.20 (1.53–3.16)	** < 0.001**
Total—per patient-year	F_res_ > ULN (n = 516)	F_res_ ≤ ULN (n = 251)		
	0.18 (0.07)	0.08 (0.04) ^†^	2.13 (1.44–3.15)	** < 0.001**

Datas are presented as means (standard error)

The number of acute exacerbation of COPD per patient-year was the number of times of exacerbation for a single patient per year

^#^After adjusting for age, sex, BMI, smoking index, smoking status, family history of respiratory diseases, occupational exposures, biomass exposure, and history of asthma, Poisson regression was applied to analyze the associations between acute exacerbations of COPD (AECOPD) in the previous year and airway impairment. CI, confidential interval. ^†^: p < 0.05; Bold values represent signifificant p values.

### Airway impairment and radiologic features

**Fig. 3:**
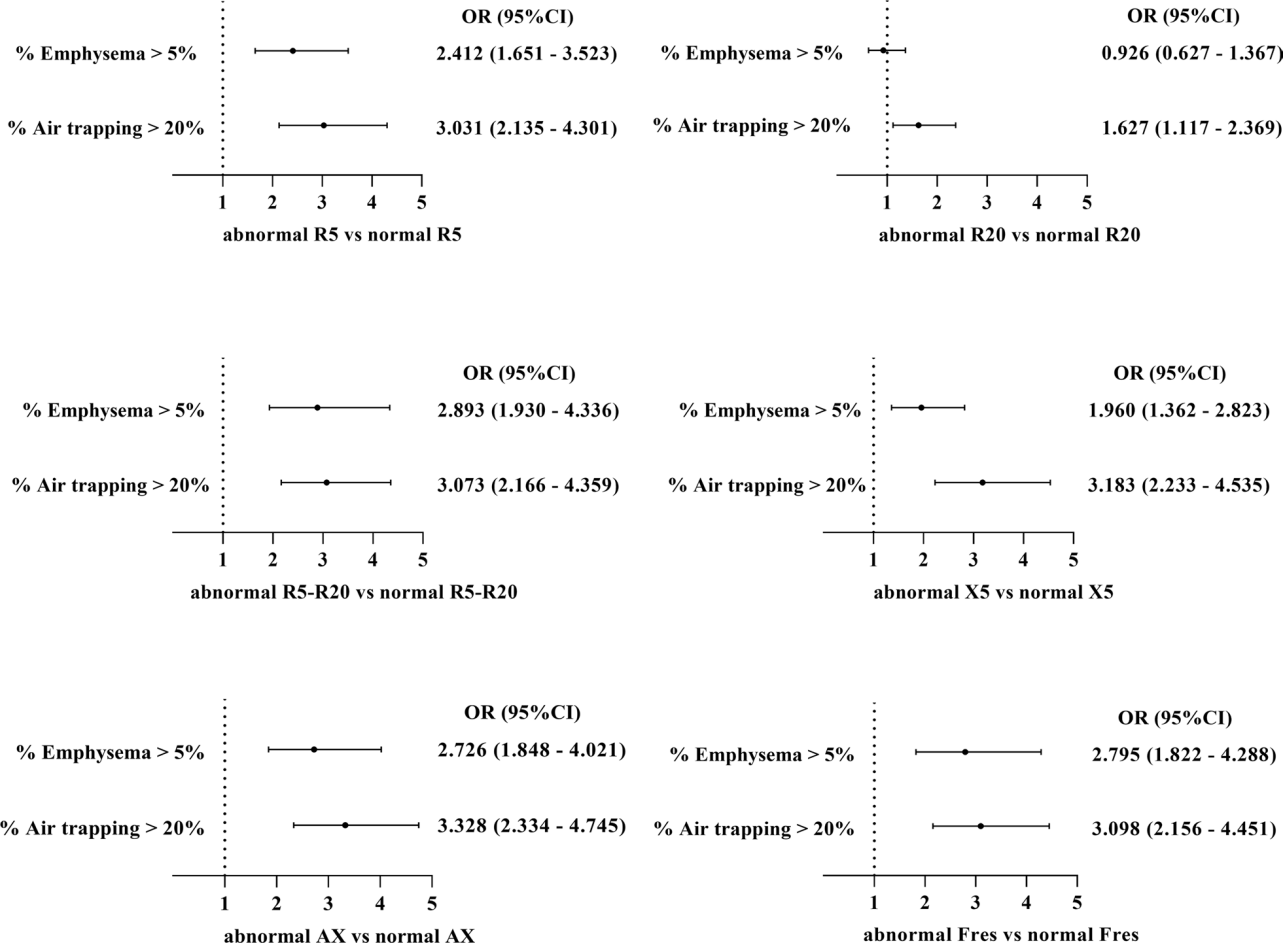
Differences in CT imaging between group with IOS parameters’ abnormalities and group with normal IOS parameters in patients with chronic obstruction pulmonary disease. OR, odds ratio; CI, confidential interval. Logistic regression analysis adjusting for age, sex, BMI, smoking index, smoking status, family history of respiratory diseases, occupational exposures, biomass exposure and history of asthma

After adjusting for age, sex, BMI, smoking status, smoking index, family history of respiratory diseases, occupational exposures, biomass exposure, and history of asthma, logistic regression analysis revealed that patients with IOS parameters’ abnormalities (R_5_, R_5_–R_20_, X_5_, AX, and F_res_) had more emphysema and air trapping than patients with normal IOS parameters (Fig. 3).

### Sensitivity analysis

Post-bronchodilator FEV_1_/FVC < 0.70 was used to define COPD in the 833 COPD subjects. Compared with patients with normal IOS parameters, patients with IOS parameters’ abnormalities had more severe respiratory symptoms, emphysema and air trapping (Additional file 1: Table S1). Likewise, patients with IOS parameters’ abnormalities had more impaired lung function and severe airway obstruction (Additional file 1: Table S2) and higher mMRC and CAT scores (Additional file 1: Fig. S1) than those with normal IOS parameters. The proportion of airway impairment in different GOLD grades was similar to that of COPD defined by post-bronchodilator FEV_1_/FVC < LLN (Additional file 1: Fig. S2). Indeed, they were similar to the results of COPD defined by post-bronchodilator FEV_1_/FVC < LLN in the association between airway impairment, AECOPD, and imaging (Additional file 1: Table S3, Fig. S3).

## Discussion

Firstly, this study indicated that airway impairment, as assessed by IOS, was present across all severities of COPD, particularly in COPD patients with GOLD 3–4. The proportion of airway impairment tended to increase with the severities of COPD, highlighting the contribution of airway impairment in COPD patients and directly linking the presence of airway impairment with COPD severities evaluated by FEV_1_. Secondly, patients with IOS parameters’ abnormalities assessed by R_5_, R_5_–R_20_, X_5_, AX, and F_res_ had more respiratory symptoms, more severe airway obstruction, more imaging structural changes, and were at a higher risk of AECOPD in the previous year than patients with normal IOS parameters. Altogether, this implies that airway impairment may be a COPD phenotype.

Herein, it was found that the proportion of airway impairment assessed by R_5_, R_5_–R_20_, X_5_, AX, and F_res_ ranged from 52.9 to 67.3% in patients with COPD. The proportion of airway impairment in our study was smaller than that in previous studies. For example, the ECLIPSE cohort results showed that the proportion of airway impairment by R_5_–R_20_, X_5_, and AX was 60%, 66% and 71% [8]. Meanwhile, Ernesto Crisafulli et al. [11] found that 74% of patients with COPD had small airway dysfunction (SAD) defined by R_5_–R_20_ > 0.07 kPa/L/s. In another study, Williamson PA et al. [26] used R_5_–R_20_ > 0.03 kPa/L/s to assess SAD, which accounted for 80% of patients with moderate COPD. The reason for these differences may be attributed to the fact that (1) there were many COPD patients with GOLD 1–2 in our study, and only about 1/3 of subjects exhibited airway impairment in COPD patients with GOLD 1. (2) Airway impairment assessed by IOS parameters > ULN or < LLN may lead to lower proportion than a fixed value. Overall, most COPD patients had airway impairment assessed by IOS, and our results also established that small airways were the chief sites of airflow obstruction in COPD [3, 27].

We further described the proportion of airway impairment across the different severities of COPD. Our results found that airway impairment was present across severities of COPD patients, particularly in GOLD 3–4 patients. The proportion of airway impairment assessed by R_5_, R_5_-R_20_, X_5_, AX, and F_res_ tended to increase with severities of COPD. This result implied that the wider airways were affected by pathophysiological abnormalities with the progression of airway obstruction. An increase in SAD parameters (R_5_, R_5_-R_20_, X_5_, AX, and F_res_) may reflect the worsening airway obstruction in COPD. Herein, significant differences were noted in airway impairment among COPD patients with GOLD 1–3, but no difference in patients with GOLD 3–4. Our result was consistent with the findings of Ana Maria G.T. Di Mango’s study [12]. This indicated IOS parameters’ abnormalities can detect initial airway obstruction changes in COPD. In addition, we found that R_20_ was not associated with the severities of COPD, given that total airway resistance increased with an increase in airway obstruction, which mainly occurred at lower frequencies rather than higher frequencies. Ana Maria G.T. Di Mango et al. reported that resistance at 16–32 Hz had no significant difference among different COPD grades [12], which may be related to the effect of upper airway shunt [21].

The present study comprehensively explored the clinical significance of airway impairment in COPD patients. The majority of studies have focused only on the clinical significance of SAD defined by R_5_–R_20_ > 0.07 kPa/s/L in chronic respiratory diseases, especially in asthma patients. However, the clinical significance of airway impairment in COPD was unclear. Our study further demonstrated that the group with IOS parameters’ abnormalities had more severe respiratory symptoms than that group with normal IOS parameters. This result was in line with the observations of Akane Haruna’s study in that SAD parameters were significantly associated with health status and dyspnea in COPD [28]. It may be that the group with IOS parameters’ abnormalities had worse lung function, more airflow limitation and imaging structural changes. Interestingly, our results found that large airway impairment assessed by R_20_ was associated with occupational exposure. To the best of our knowledge, this finding has not been described in previous studies. Some studies determined that occupational exposure to endotoxin and exposure to vapors, gas, dust, or fumes were associated with large airways (wall area percent) [29, 30]. Our result also adds evidence to this view from the perspective of respiratory mechanics. Larger airway size may lower the ability of the respiratory defense mechanisms to remove harmful substances [31]. In addition, peripheral airway impairment assessed by F_res_ was associated with chronic bronchitis, which had the excessive secretion of mucus, and resulted in small airway obstruction [32, 33].

AECOPD were the predominant reason for hospitalization and mortality in COPD. Moreover, AECOPD in the previous year were independent predictor of AECOPD in the following year [34]. Additionally, our study showed that airway impairment was associated with the risk of AECOPD in the previous year. This finding is of great clinical significance in COPD patients; this signals that COPD patients with airway impairment may have a poorer prognosis and may be a special COPD subtype. AECOPD are highly implicated in airway remodeling, thereby leading to airway lesions [35–39]. This view was validated by prior studies that reported that AECOPD patients had aberrant airway resistance and increased annual change in airway resistance [40, 41]. Therefore, it is crucial to promptly identify and treat airway impairment; this will eventually minimize the risk of AECOPD as well as the economic burden of COPD patients. Omar S. Usmani et al. proposed approaches to optimize small airway treatment of COPD, including optimized drug formulations, inhalers, and drugs to improve small airways [42].

There were some limitations in our study that need to be considered when interpreting the results. Firstly, there are many COPD patients with GOLD 1–2, which may impact the proportion of airway impairment. However, our study still demonstrated that airway impairment was present across patients with all severities of COPD. Secondly, the proportion and clinical characteristics of airway impairment in GOLD ABCD or ABE group were not shown in our study, because this study was community-based study. Patients in our study had less symptoms and AECOPD than those in hospital-based study. Meanwhile, FEV_1_ measured by body plethysmograph instead of spirometry was more accurate in reflecting COPD severity grade [43]. Finally, there were some differences between the forced oscillation technique (FOT) and IOS in instrument characteristics, oscillating signal and data post-processing; FOT may be more sensitive to measuring reactance in patients with airflow obstruction than IOS [44].

## Conclusion

To conclude, airway impairment was present across patients with severities of COPD, particularly in GOLD 3–4 COPD patients. Airway impairment assessed by R_5_, R_5_–R_20_, X_5_, AX, and F_res_ had more severe respiratory symptoms, airway obstruction, imaging structural changes, and a higher risk of AECOPD in the previous years. Our study revealed that airway impairment may potentially be a phenotype of COPD. Further studies are warranted to identify underlying mechanisms and longitudinal progression of airway impairment.

## Supplementary Information

**Additional file 1. Table S1.** Baseline characteristics airway impairment assessed by IOS in patients with chronic obstructionPulmonary disease (post-bronchodilator FEV_1_/FVC < 0.7). **Table S2.** Post-bronchodilator lung function and pre-bronchodilator IOS parameters of airway impairment in patients with chronic obstruction pulmonary disease (post-bronchodilator FEV_1_/FVC < 0.70). **Table S3.** Associations between acute exacerbations of COPD in the previous year and airway impairment in patients with chronic obstruction pulmonary disease. (post-bronchodilator FEV_1_/FVC < 0.70). **Figure S1.** mMRC and CAT scores between airway impairment group and non-airway impairment group in patients with chronic obstruction pulmonary disease (post-bronchodilator FEV_1_/FVC < 0.7). **Figure S2.** Proportion of airway impairment in patients with chronic obstruction pulmonary disease (post-bronchodilator FEV_1_/FVC < 0.70) with different GOLD grades. **Figure S3.** Differences in CT imaging between group with IOS parameters’ abnormalities and group with normal IOS parameters in patients with chronic obstruction pulmonary disease (post-bronchodilator FEV_1_/FVC < 0.70).

## Data Availability

The datasets used and/or analyzed during the current study are available from the corresponding author on reasonable request.

## Abbreviations

SAD: Small airway dysfunction
COPD: Chronic obstructive pulmonary disease
MMEF: Maximum mid expiratory flow
FEV_1_: Forced expiratory volume in one second
FVC: Forced vital capacity
CT: Computed tomography
IOS: Impulse oscillometry
R_5_-R_20_: Difference from R_5_ to R_20_
R_5_: Resistance at 5 Hz
R_20_: Resistance at 20 Hz
X_5_: Reactance at 5 Hz
F_res_: Resonant frequency
OR: Odds ratio
RR: Risk ratio
CI: Confidential interval
BMI: Body mass index
