# Supplementary material for: Clinical characteristics of airway impairment assessed by impulse oscillometry in patients with chronic obstructive pulmonary disease: findings from the ECOPD study in China
Source: BMC Pulm Med. 2023 Feb 3;23:52. doi: 10.1186/s12890-023-02311-z (PMC9896683; doi:10.1186/s12890-023-02311-z)
Supplement: Supplementary file 1 — Additional file 1. Table S1. Baseline characteristics airway impairment assessed by IOS in patients with chronic obstructionPulmonary disease (post-bronchodilator FEV1/FVC < 0.7). Table S2. Post-bronchodilator lung function and pre-bronchodilator IOS parameters of airway impairment in patients with chronic obstruction pulmonary disease (post-bronchodilator FEV1/FVC < 0.70). Table S3. Associations between acute exacerbations of COPD in the previous year and airway impairment in patients with chronic obstruction pulmonary disease. (post-bronchodilator FEV1/FVC < 0.70). Figure S1. mMRC and CAT scores between airway impairment group and non-airway impairment group in patients with chronic obstruction pulmonary disease (post-bronchodilator FEV1/FVC < 0.7). Figure S2. Proportion of airway impairment in patients with chronic obstruction pulmonary disease (post-bronchodilator FEV1/FVC < 0.70) with different GOLD grades. Figure S3. Differences in CT imaging between group with IOS parameters’ abnormalities and group with normal IOS parameters in patients with chronic obstruction pulmonary disease (post-bronchodilator FEV1/FVC < 0.70). [file 12890_2023_2311_MOESM1_ESM.docx]

**Table S1** Baseline characteristics of airway impairment assessed by IOS in patients with chronic obstruction pulmonary disease. (post-bronchodilator FEV_1_/FVC < 0.7)

|  | R_5_ | | R_20_ | | R_5_-R_20_ | | X_5_ | | AX | | F_res_ | | |
| --- | --- | --- | --- | --- | --- | --- | --- | --- | --- | --- | --- | --- | --- |
| Value | ≤ ULN | > ULN | ≤ ULN | > ULN | ≤ ULN | > ULN | ≤ ULN | > ULN | ≤ ULN | > ULN | ≤ ULN | > ULN |  |
| Number, n (%) | 352 (42.3) | 481 (57.7) | 596 (71.5) | 237 (28.5) | 322 (38.7) | 511 (61.3) | 406 (48.7) | 427 (51.3) | 337 (40.5) | 496 (59.5) | 276 (33.1) | 557 (66.9) |  |
| Age | 64.59 (7.04) | 64.78 (7.15) | 64.83 (6.89) | 64.38 (7.61) | 63.51 (6.93) | 65.45 (7.11) * | 63.75 (6.99) | 65.60 (7.10) * | 63.64 (6.94) | 65.42 (7.12) * | 62.88 (7.01) | 65.60 (6.98) * |  |
| Male, n (%) | 328 (93.2) | 435 (90.4) | 548 (91.9) | 215 (90.7) | 290 (90.1) | 473 (92.6) | 381 (93.8) | 382 (89.5) ^†^ | 316 (93.8) | 447 (90.1) | 252 (91.3) | 511 (91.7) |  |
| BMI | 22.23 (2.98) | 21.94 (3.38) | 21.95 (3.16) | 22.36 (3.36) | 22.41 (3.13) | 21.85 (3.26) ^†^ | 22.21 (2.99) | 21.92 (3.42) | 22.20 (3.07) | 21.97 (3.32) | 22.46 (3.21) | 21.87 (3.21) ^†^ |  |
| Smoking statue, n (%) |  |  |  |  |  |  |  |  |  |  |  |  |  |
| Never | 46 (13.1) | 64 (13.3) | 77 (12.9) | 33 (13.9) | 49 (15.2) | 61 (11.9) | 50 (12.3) | 60 (14.1) | 43 (12.8) | 67 (13.5) | 41 (14.9) | 69 (12.4) |  |
| Ever | 91 (25.9) | 162 (33.7) ^†^ | 170 (28.5) | 83 (35.0) | 79 (24.5) | 174 (34.1) ^†^ | 107 (26.4) | 146 (34.2) ^†^ | 85 (25.2) | 168 (33.9) ^†^ | 71 (25.7) | 182 (32.7) ^†^ |  |
| Current | 215 (61.1) | 255 (53.0) ^†^ | 349 (58.6) | 121 (51.1) ^†^ | 194 (60.2) | 276 (54.0) | 249 (61.3) | 221 (51.8) ^†^ | 209 (62.0) | 261 (52.6) ^†^ | 164 (59.4) | 306 (54.9) |  |
| Pack-years | 35.91 (30.80) | 38.12 (32.96) | 37.22 (31.60) | 37.12 (33.27) | 35.05 (31.33) | 38.54 (32.48) | 35.86 (30.80) | 38.46 (33.20) | 35.12 (30.54) | 38.59 (33.01) | 34.31 (30.31) | 38.62 (32.83) |  |
| Family history of respiratory diseases, n (%) | 61 (17.3) | 93 (19.3) | 107 (18.0) | 47 (19.8) | 57 (17.7) | 97 (19.0) | 75 (18.5) | 79 (18.5) | 57 (16.9) | 97 (19.6) | 44 (15.9) | 110 (19.7) |  |
| Occupational exposures, n (%) | 86 (24.4) | 127 (26.4) | 136 (22.8) | 77 (32.5) ^†^ | 80 (24.8) | 133 (26.0) | 115 (28.3) | 98 (23.0) | 87 (25.8) | 126 (25.4) | 67 (24.3) | 146 (26.2) |  |
| Biomass exposure, n (%) | 122 (34.7) | 190 (39.5) | 223 (37.4) | 89 (37.6) | 111 (34.5) | 201 (39.3) | 145 (35.7) | 167 (39.1) | 115 (34.1) | 197 (39.7) | 93 (33.7) | 219 (39.3) |  |
| History of CB, n (%) | 19 (5.4) | 42 (8.7) | 44 (7.4) | 17 (7.2) | 19 (5.9) | 42 (8.2) | 25 (6.2) | 36 (8.4) | 20 (5.9) | 41 (8.3) | 12 (4.3) | 49 (8.8) ^†^ |  |
| History of asthma, n (%) | 2 (0.6) | 20 (4.2) ^†^ | 11 (1.8) | 11 (4.6) ^†^ | 2 (0.6) | 20 (3.9) ^†^ | 1 (0.2) | 21 (4.9) * | 1 (0.3) | 21 (4.2) ^†^ | 1 (0.4) | 21 (3.8) ^†^ |  |
| Drug treatment, n (%) | 142 (40.3) | 250 (52.0) ^†^ | 271 (45.5) | 121 (51.1) | 124 (38.5) | 268 (52.4) * | 164 (40.4) | 228 (53.4) * | 136 (40.4) | 256 (51.6) ^†^ | 106 (38.4) | 286 (51.3) * |  |
| Clinical symptoms, (n%) |  |  |  |  |  |  |  |  |  |  |  |  |  |
| Cough | 117 (33.2) | 224 (46.6) * | 239 (40.1) | 102 (43.0) | 103 (32.0) | 238 (46.6) * | 140 (34.5) | 201 (47.1) * | 112 (33.2) | 229 (46.2) * | 85 (30.8) | 256 (46.0) * |  |
| Phlegm | 135 (38.4) | 274 (57.0) * | 284 (47.7) | 125 (52.7) | 124 (38.5) | 285 (55.8) * | 164 (40.4) | 245 (57.4) * | 129 (38.3) | 280 (56.5) * | 101 (36.6) | 308 (55.3) * |  |
| Wheeze | 36 (10.2) | 114 (23.7) * | 97 (16.3) | 53 (22.4) ^†^ | 33 (10.2) | 117 (22.9) * | 43 (10.6) | 107 (25.1) * | 33 (9.8) | 117 (23.6) * | 27 (9.8) | 123 (22.1) * |  |
| Dyspnea | 103 (29.3) | 241 (50.2) * | 235 (39.4) | 109 (46.2) | 87 (27.0) | 257 (50.4) * | 119 (29.3) | 225 (52.8) * | 95 (28.2) | 249 (50.3) * | 72 (26.1) | 272 (48.9) * |  |
| GOLD stage, n (%) |  |  |  |  |  |  |  |  |  |  |  |  |  |
| GOLD 1 | 245 (69.6) | 122 (25.4) * | 289 (48.5) | 78 (32.9) * | 236 (73.3) | 131 (25.6) * | 264 (65.0) | 103 (24.1) * | 244 (72.4) | 123 (24.8) * | 211 (76.4) | 156 (28.0) * |  |
| GOLD 2 | 100 (28.4) | 262 (54.5) * | 234 (39.3) | 128 (54.0) * | 82 (25.5) | 280 (54.8) * | 131 (32.3) | 231 (54.1) * | 87 (25.8) | 275 (55.4) * | 62 (22.5) | 300 (53.9) * |  |
| GOLD 3 | 7 (2.0) | 81 (16.8) * | 62 (10.4) | 26 (11.0) | 4 (1.2) | 84 (16.4) * | 11 (2.7) | 77 (18.0) * | 6 (1.8) | 82 (16.5) * | 3 (1.1) | 85 (15.3) * |  |
| GOLD 4 | 0 (0) | 16 (3.3) * | 11 (1.8) | 5 (2.1) | 0 (0) | 16 (3.1) * | 0 (0) | 16 (3.7) * | 0 (0) | 16 (3.2) * | 0 (0) | 16 (2.9) * |  |
| Emphysema on CT, (%) | 65 (18.5) | 170 (35.3) * | 169 (28.4) | 66 (27.8) | 49 (15.2) | 186 (36.4) * | 83 (20.4) | 152 (35.6) * | 58 (17.2) | 177 (35.7) * | 43 (15.6) | 192 (34.5) * |  |
| Air trapping on CT, (%) | 151 (42.9) | 317 (65.9) * | 321 (53.9) | 147 (62.0) ^†^ | 129 (40.1) | 339 (66.3) * | 179 (44.1) | 289 (67.7) * | 138 (40.9) | 330 (66.5) * | 106 (38.4) | 362 (65.0) * |  |
|  |  |  |  |  |  |  |  |  |  |  |  |  |  |

Datas are presented as the mean (standard deviation) or median (interquartile range) and were analyzed by Student’s t-test or Wilcoxon’s rank-sum test.; BMI, body mass index; MMEF, maximum mid expiratory flow, MMEF; FEV_1_, forced expiratory volume in one second; CT, computed tomography. ^†^: P < 0.05; *: P <0.001

**Table S2** Post-bronchodilator lung function and pre-bronchodilator IOS parameters of airway impairment in patients with chronic obstruction pulmonary disease (post-bronchodilator FEV_1_/FVC < 0.70).

|  | R_5_ | | R_20_ | | R_5_-R_20_ | | X_5_ | | AX | | F_res_ | |
| --- | --- | --- | --- | --- | --- | --- | --- | --- | --- | --- | --- | --- |
| Value | Normal | Abnormal | Normal | Abnormal | Normal | Abnormal | Normal | Abnormal | Normal | Abnormal | Normal | Abnormal |
| Spirometry |  |  |  |  |  |  |  |  |  |  |  |  |
| FEV_1_, L | 2.30 (0.50) | 1.73 (0.56) * | 2.03 (0.61) | 1.83 (0.56) * | 2.33 (0.51) | 1.75 (0.55) * | 2.29 (0.53) | 1.67 (0.51) * | 2.36 (0.48) | 1.71 (0.53) * | 2.40 (0.49) | 1.77 (0.55) * |
| FEV_1_, %predicted | 86.70 (15.49) | 66.53 (18.26) * | 76.92 (20.29) | 70.36 (17.79) * | 88.14 (14.56) | 66.81 (18.21) * | 85.14 (16.17) | 65.46 (18.16) * | 87.92 (14.74) | 66.31 (18.00) * | 89.42 (14.44) | 67.93 (18.21) * |
| FVC, L | 3.66 (0.72) | 3.14 (0.72) * | 3.41 (0.77) | 3.21 (0.72) * | 3.68 (0.73) | 3.15 (0.71) * | 3.68 (0.72) | 3.04 (0.66) * | 3.75 (0.69) | 3.09 (0.70) * | 3.77 (0.71) | 3.15 (0.71) * |
| FVC, %predicted | 108.59 (17.23) | 95.29 (17.02) * | 102.13 (18.77) | 97.85 (16.78) ^†^ | 110.45 (16.37) | 94.90 (16.89) * | 108.29 (16.55) | 93.89 (17.14) * | 110.31 (16.41) | 94.52 (16.74) * | 111.50 (16.37) | 95.66 (16.92) * |
| FEV_1_/FVC, L | 63.02 (6.09) | 54.86 (10.64) * | 58.94 (9.70) | 56.72 (10.08) ^†^ | 63.35 (5.88) | 55.13 (10.52) * | 62.08 (6.87) | 54.72 (10.88) * | 63.08 (5.94) | 55.06 (10.64) * | 63.69 (5.42) | 55.64 (10.46) * |
| FEF_50_, %predicted | 39.32 (13.28) | 25.18 (12.65) * | 32.29 (15.07) | 28.27 (13.25) * | 40.20 (13.32) | 25.46 (12.48) * | 38.12 (13.98) | 24.50 (12.02) * | 40.17 (13.28) | 25.03 (12.23) * | 41.48 (12.83) | 26.04 (12.73) * |
| FEF_75_, %predicted | 26.62 (10.35) | 20.17 (8.60) * | 23.19 (10.14) | 22.12 (9.24) | 26.68 (10.17) | 20.51 (8.94) * | 25.95 (10.33) | 19.97 (8.51) * | 26.77 (10.55) | 20.26 (8.49) * | 27.05 (10.13) | 20.84 (9.12) * |
| MMEF, %predicted | 34.26 (10.82) | 23.32 (10.57) * | 28.75 (12.17) | 25.87 (11.19) ^†^ | 34.81 (11.00) | 23.63 (10.44) * | 33.33 (11.48) | 22.80 (10.00) * | 34.88 (11.07) | 23.24 (10.13) * | 35.72 (10.63) | 24.10 (10.66) * |
| IOS |  |  |  |  |  |  |  |  |  |  |  |  |
| R_5_, kPa/L/s | 0.26 (0.23 – 0.29) | 0.42 (0.36 – 0.51) * | 0.31 (0.25 – 0.39) | 0.44 (0.38 – 0.54) * | 0.27 (0.24 – 0.32) | 0.41 (0.33 – 0.49) * | 0.28 (0.24 – 0.34) | 0.42 (0.35 – 0.52) * | 0.26 (0.23 – 0.32) | 0.41 (0.34 – 0.50) * | 0.26 (0.23 – 0.32) | 0.40 (0.32 – 0.48) * |
| R_20_, kPa/L/s | 0.23 (0.20 – 0.25) | 0.30 (0.26 – 0.34) * | 0.24 (0.22 – 0.27) | 0.33 (0.31 – 0.37) * | 0.25 (0.22 – 0.30) | 0.27 (0.24 – 0.32) * | 0.25 (0.21 – 0.29) | 0.28 (0.25 – 0.33) * | 0.24 (0.21 – 0.29) | 0.28 (0.24 – 0.32) * | 0.24 (0.21 – 0.29) | 0.27 (0.24 – 0.32) * |
| R_5_-R_20_, kPa/L/s | 0.03 (0.01 – 0.05) | 0.13 (0.08 – 0.19) * | 0.06 (0.03 – 0.12) | 0.10 (0.05 – 0.18) * | 0.02 (0.01 – 0.04) | 0.12 (0.08 – 0.18) * | 0.04 (0.02 – 0.06) | 0.13 (0.08 – 0.20) * | 0.03 (0.01 – 0.04) | 0.12 (0.08 – 0.19) * | 0.02 (0.01 – 0.04) | 0.11 (0.07 – 0.17) * |
| X_5_, kPa/L/s | -0.09 (-0.11 – 0.07) | -0.17 (-0.24 – 0.12) * | -0.11 (-0.16 – 0.08) | -0.16 (-0.24 – 0.10) * | -0.09 (-0.11 – 0.07) | -0.16 (-0.23 – 0.11) * | -0.08 (-0.10 – 0.07) | -0.18 (-0.25 – 0.14) * | -0.08 (-0.10 – 0.07) | -0.16 (-0.24 – 0.12) * | -0.08 (-0.10 – 0.07) | -0.15 (-0.22 – 0.11) * |
| AX, kPa/L | 0.24 (0.15 – 0.40) | 1.22 (0.64 – 2.24) * | 0.47 (0.21 – 1.13) | 1.07 (0.42 – 2.35) * | 0.21 (0.13 – 0.32) | 1.15 (0.60 – 2.16) * | 0.25 (0.15 – 0.41) | 1.40 (0.78 – 2.35) * | 0.21 (0.14 – 0.30) | 1.21 (0.69 – 2.18) * | 0.18 (0.12 – 0.27) | 1.02 (0.55 – 2.01) * |
| F_res_, HZ | 12.52 (9.65 – 15.29) | 21.33 (17.22 – 25.12) * | 15.87 (11.57 – 20.92) | 20.30 (14.37 – 26.28) * | 11.40 (9.31 – 13.80) | 21.05 (17.14 – 24.88) * | 12.86 (9.86 – 15.95) | 21.55 (17.73 – 25.38) * | 11.47 (9.37 – 13.56) | 21.28 (17.78 – 24.95) * | 10.62 (9.12 – 12.58) | 20.42 (16.70 – 24.49) * |

Datas are presented as the mean (standard deviation) or median (interquartile range) and were analyzed by Student’s t-test or Wilcoxon’s rank-sum test; IOS, impulse oscillometry; R_5_, resistance at 5 Hz; R_20_, resistance at 20 Hz; R_5_-R_20_, difference from R_5_ to R_20_; X_5_, reactance at 5 Hz; F_res_, resonant frequency. FVC, forced vital capacity; MMEF, maximal mid-expiratory flow; FEF_50_, forced expiratory flow 50%; FEF_75_, forced expiratory flow 75%. ^†^: P < 0.05; *: P <0.001.

**Table S3** Associations between acute exacerbations of COPD in the previous year and airway impairment in patients with chronic obstruction pulmonary disease. (post-bronchodilator FEV_1_/FVC < 0.70).

| AECOPD in the previous year | With airway impairment | Without airway impairment | Risk Ratio (95% CI) ^#^ | P Value |
| --- | --- | --- | --- | --- |
| Total — per patient-year | R_5_ > ULN (n = 481) | R_5_ ≤ ULN (n = 351) |  |  |
|  | 0.17 (0.06) | 0.11 (0.05) | 1.48 (1.08 - 2.02) | **0.014** |
| Total — per patient-year | R_20_ > ULN (n = 237) | R_20_ ≤ ULN (n = 595) |  |  |
|  | 0.15 (0.06) | 0.17 (0.06) | 0.93 (0.67 - 1.28) | 0.641 |
| Total — per patient-year | R_5_-R_20_ > ULN (n = 511) | R_5_-R_20_ ≤ ULN (n = 321) |  |  |
|  | 0.17 (0.07) | 0.11 (0.04) ^†^ | 1.61 (1.16 - 2.23) | **0.005** |
| Total — per patient-year | X_5_ < LLN (n = 427) | X_5_ ≥ LLN (n = 405) |  |  |
|  | 0.18 (0.07) | 0.09 (0.04) ^†^ | 2.05 (1.49 - 2.81) | **< 0.001** |
| Total — per patient-year | AX > ULN (n = 496) | AX ≤ ULN (n = 336) |  |  |
|  | 0.17 (0.06) | 0.08 (0.03) ^†^ | 2.04 (1.45 - 2.86) | **< 0.001** |
| Total — per patient-year | F_res_ > ULN (n = 556) | F_res_ ≤ ULN (n = 276) |  |  |
|  | 0.17 (0.06) | 0.08 (0.03) ^†^ | 2.19 (1.50 – 3.20) | **< 0.001** |

Datas are presented as means (standard error).

The number of acute exacerbation of COPD per patient-year was the number of times of exacerbation for a single patient per year.

^#^: After adjusting for age, sex, BMI, smoking index, smoking status, family history of respiratory diseases, occupational exposures, biomass exposure, and history of asthma, Poisson regression was applied to analyze the associations between acute exacerbations of COPD in the previous year and airway impairment. CI, confidential interval.  ^†^: p < 0.05;

**Figure S1** mMRC and CAT scores between airway impairment group and non-airway impairment group in patients with chronic obstruction pulmonary disease.

(post-bronchodilator FEV_1_/FVC < 0.7).

datas were shown as mean (SE).

** means <0.01, *** means <0.001

**Figure S2** Proportion of airway impairment in patients with chronic obstruction pulmonary disease (post-bronchodilator FEV_1_/FVC < 0.70) with different GOLD grades.

**Figure S3** Differences in CT imaging between group with IOS parameters’ abnormalities and group with normal IOS parameters in patients with chronic obstruction pulmonary disease (post-bronchodilator FEV_1_/FVC < 0.70).

OR, odds ratio; CI, confidential interval. Logistic regression analysis adjusting for age, sex, BMI, smoking index, smoking statue, family history of respiratory diseases, occupational exposures, biomass exposure and history of asthma.


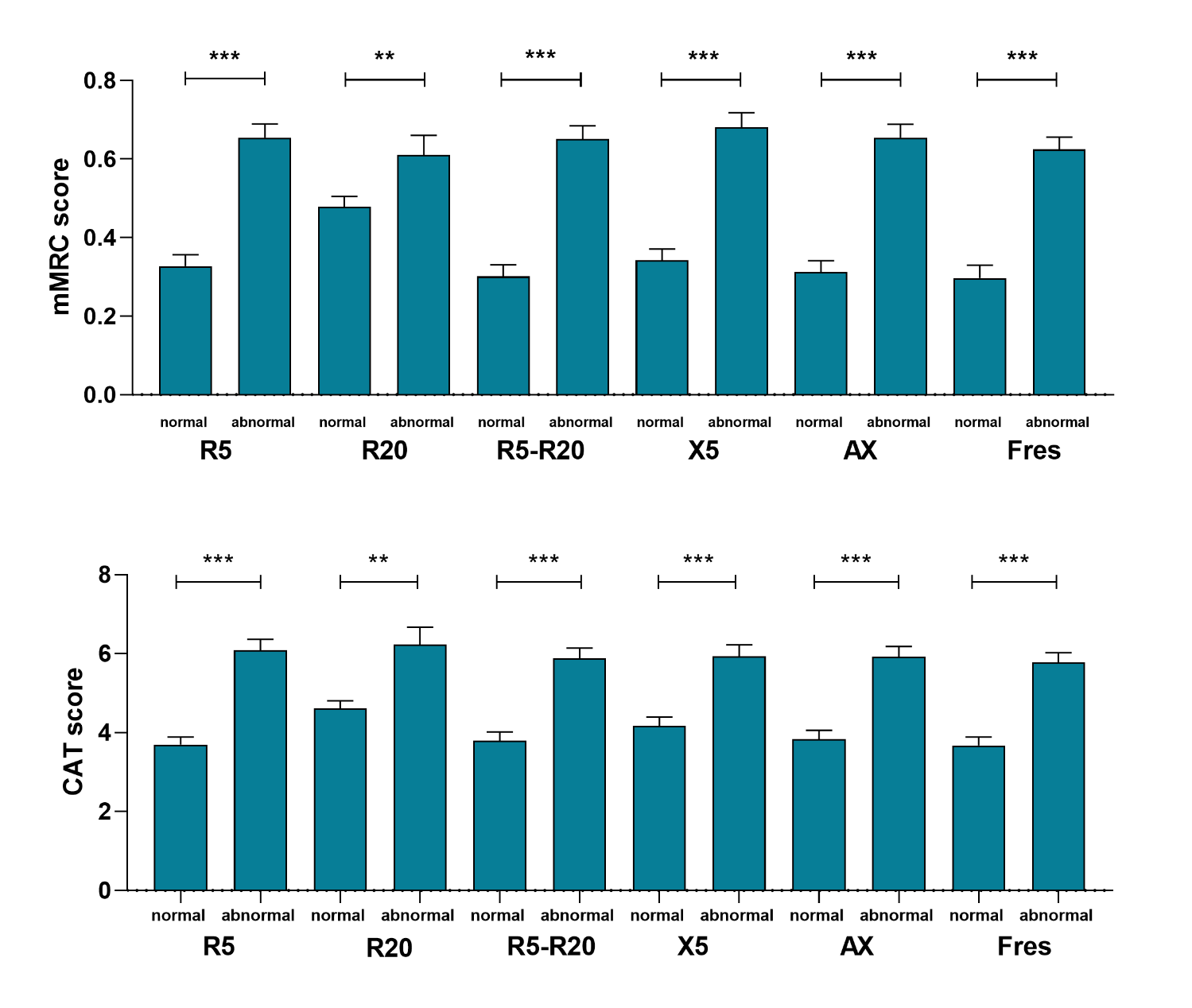
Figure S1


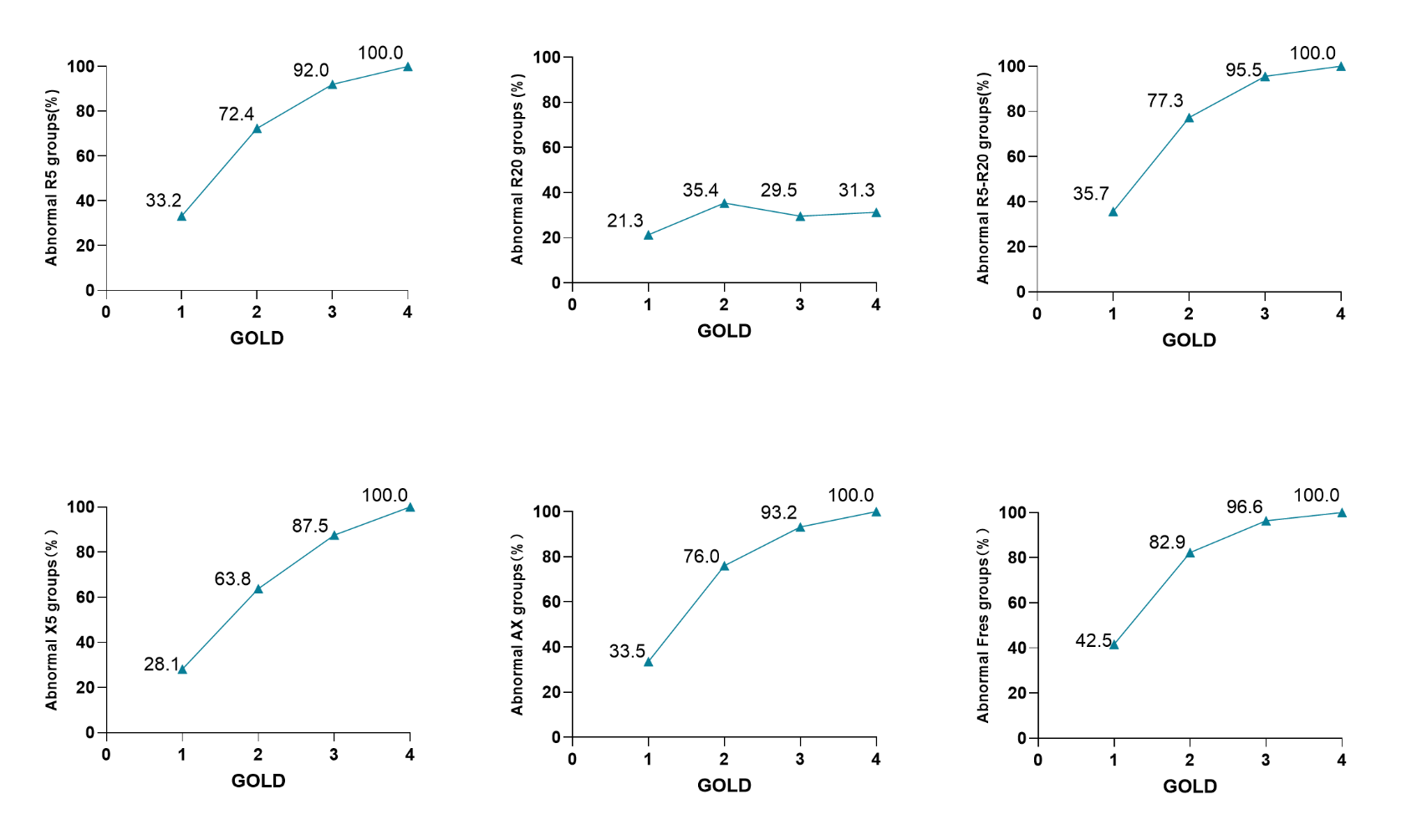
Figure S2


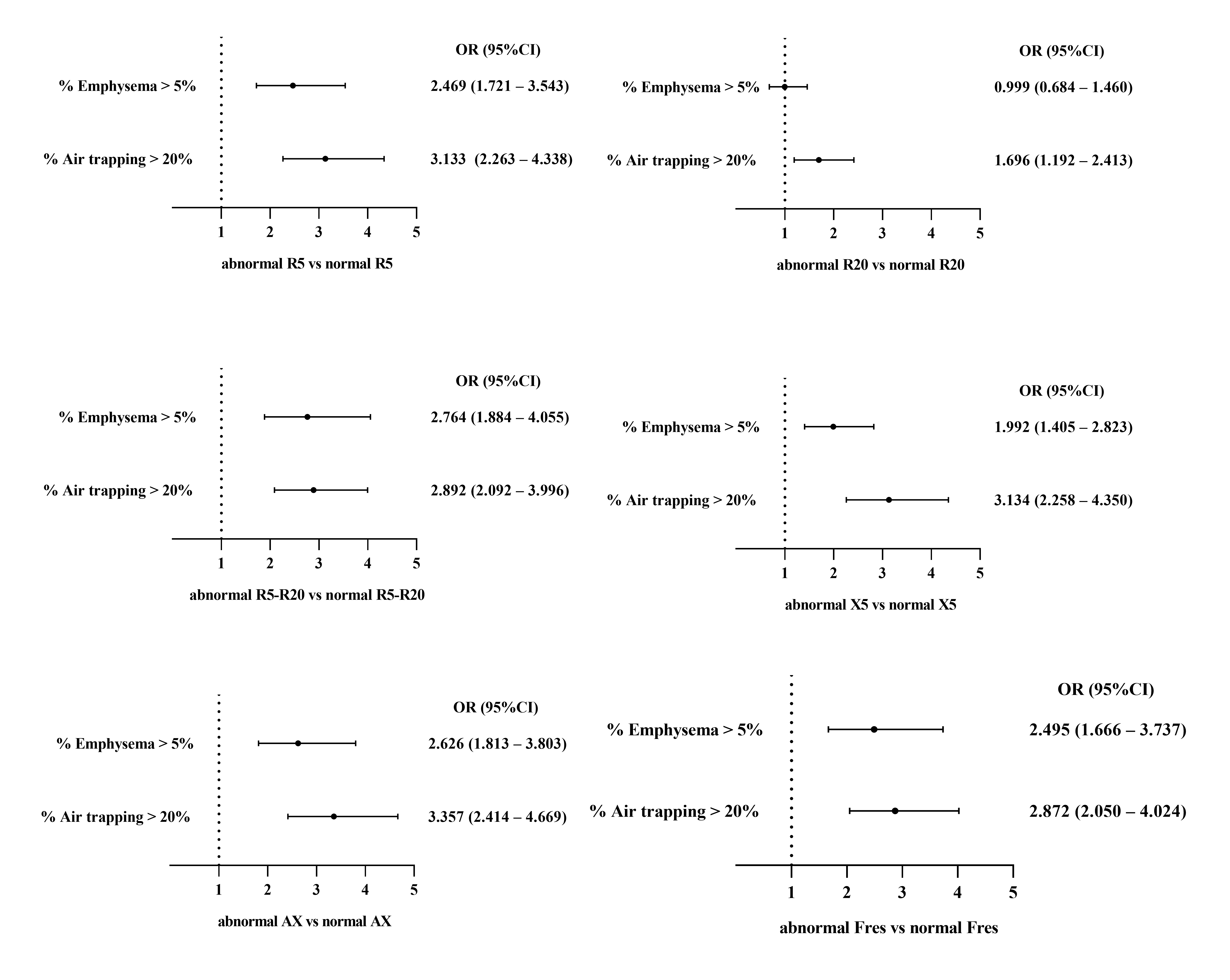
Figure S3
